# The Hospital. Nursing Section

**Published:** 1904-06-25

**Authors:** 


The Hospital.

Nursing Section.

Contributions for this Section of "The Hospital" should be addressed to the Editor, "The Hospital" Nursing Section, 28 & 29 Southampton Street, Strand, London, W.C.

No. 926.—Vol. XXXVI. *SATURDAY, JUNE 25, 1904.*

### Notes on News from the Nursing World.

### DISAPPEARANCE OF A SISTER AT RIO DE JANEIRO.

On Friday, May 6th, between 7 and 8 o'clock in the morning, Sister Isobel Greuer, one of the most highly esteemed of the sisters on the nursing staff of the Strangers' Hospital at Rio de Janeiro, left her room, stating, in answer to a question addressed to her by one of her companions, that she was going for a walk. As she had not returned at 2 P.M. when she should have gone on duty, the matron reported her absence to the hon. secretary of the hospital, who, on the following morning, as she had not then returned, communicated with the police authorities. The latter at once took steps to endeavour to discover what had become of her. She was traced to the Igrejinha Copacabana where she had been seen by two persons at about 11 o'clock on the morning of May 6th, one of these persons describing her appearance and costume very accurately. Since then no trace whatever has been found of Miss Greuer, who, it is surmised, must have either fallen, or been swept into the sea, by wind or wave, from the slippery rocks at that place. This is the conclusion of the police authorities. It was also adopted by the board of directors after a careful consideration of the circumstances at a meeting held on Wednesday, May 18th. The shore has been continuously watched for any appearance of the body or clothing, but up to the present no signs of either have been seen. Miss Greuer, it may be added, arrived at Rio in August 1903, and we understand that she was a member of the Royal National Pension Fund for Nurses.

### THE QUEEN'S NURSES IN SCOTLAND.

One of the most interesting papers at the International Home Relief Congress at Edinburgh was read by Miss Guthrie Wright, honorary secretary of the Scottish Branch of Queen Victoria's Jubilee Institute for Nurses. Miss Wright stated that during the last reported year of the 9,801 cases admitted to the Edinburgh Royal Infirmary, 5,592 were inhabitants of Edinburgh and Leith, and during a corresponding period 5,341 persons were nursed in their own homes in the city and the burgh by the Institute's district nurses. She contended that there is a large number of persons, chiefly women, who are willing to receive skilled nursing at home, but who will not, or cannot, go to hospitals; and she claims that the Queen's nurses supply the needs of these. At the same Congress, Dr. Van Deventer, of Haarlem, urged the necessity for special training in the case of nurses tending patients in their own homes, and this is given under the auspices of the Jubilee Institute.

### QUEEN ALEXANDRA'S MILITARY NURSING SERVICE.

Miss B. Rennie has been appointed staff nurse in Queen Alexandra's Imperial Military Nursing Service, and has been posted to the Royal Victoria Hospital, Netley. Sister J. M. Clay and Staff Nurses M. L. Harris, E. J. M. Keene, and F. E. C. Watson have been confirmed in their appointments, their periods of provisional service having expired. Sister A. Cameron has retired on pension. Sister Lamming, of the Royal Herbert Hospital, Woolwich, has been transferred to the Cadets' Hospital, Royal Military Academy, for temporary duty, Matron G. M. Richards from Woolwich to Devonport, Matron M. Wilson from Aldershot to Woolwich, and Sister M. L. Potter from Hounslow to Dublin.

### HEROISM OF AMERICAN NURSES.

A pleasing feature in connection with the terrible disaster to the steamer *General Slocum* in New York Harbour is the heroism of American nurses. It is stated that several of the nurses employed at the hospital on North Brother Island both swam and waded out to the vessel and saved many lives. One of these, Miss Macdonald, is credited with having brought to land eight living children, and afterwards taken seven of the dead out of the water.

### THE WOLVERHAMPTON NURSING INSTITUTION AND THE PENSION FUND.

At the annual meeting of the Queen Victoria Nursing Institution at Wolverhampton last week it was announced that a new departure had been taken with the object of benefiting the staff. In order to provide an inducement for nurses to remain at the Home the Committee have adopted a scheme of affiliation with the Royal National Pension Fund. Accordingly, with the view of assisting nurses under 40 who may desire to join the Fund, the Committee pay an annual sum to it, not exceeding £6 a nurse. Several nurses have already joined, and the help thus given is approved by the subscribers to the Institution. Another feature of the report is the statement that in the past six years the Committee advanced £2,321 to the district branch. The grant last year was £145. In addition, the mortgage was reduced to the extent of £200, and £100 was transferred to the Renovation and Improvements Fund. The valuable services of Miss Loveys, the matron of the Institution are warmly acknowledged in the report, and were not overlooked at the meeting.

### THE RELIGIOUS QUESTION IN NURSING.

It is curious that at a moment when the Roman Catholic laity and clergy are protesting that nurses of their faith are not on an equality with other nurses in certain English hospitals, the trustees of the late Marquis of Bute should persist in the view that the nursing at the Cardiff Seamen's Hospital ought to be entrusted to a Roman Catholic Sisterhood. The other day a deputation from Cardiff waited upon the trustees in London, and this question was discussed at length. The trustees in order to try and convince the deputation that they were right, took the latter to the Italian Hospital, where the nursing is done by Roman Catholic Sisters, and where the managers are of all creeds. But the deputation, in spite of this object-lesson, went unsatisfied away, and we sympathise with them. Canon Keatinge, in his sermon at the Southwark Roman Catholic Cathedral on Sunday morning, maintained that Roman Catholics should not be required to take part in the public prayers in the wards of hospitals which are not in consonance with their convictions; and we recognise that here there may be a grievance. But if Roman Catholics desire to have their grievances removed, they should be willing to give as well as take. The Seamen's Hospital at Cardiff must, in the nature of things, mainly depend upon the contributions of the residents, the majority of whom are either members of the Church of England or Nonconformists, who would strongly object to support an institution in which the nursing was entrusted to a Roman Catholic sisterhood. As the late Lord Bute's will only provides that this arrangement should be made "if possible," we maintain that the trustees ought to be perfectly satisfied if it is agreed that Roman Catholic nurses shall be adequately represented on the staff. It is much to be regretted that the question of religious tests should be always cropping up, and large-hearted people, whether Romanists or Protestants, would do the cause of suffering humanity a great service if they addressed themselves to the task of settlement by mutual concessions.

### POPLAR AND THE WORLD.

It being announced at the last meeting of managers of the Poplar and Stepney Sick Asylum that one of the nurses had obtained an appointment in Egypt, one of the managers observed, "We train nurses for the whole world." At the same meeting the Visiting Committee reported, having considered the vacancy in the office of first assistant matron, consequent upon the resignation of Miss Smith, who has been appointed matron of the Charlton Infirmary, recommended that, subject to the approval of the Local Government Board, Miss Alice Florence Foyster, second assistant matron, be promoted to fill the vacancy, at a commencing salary of £50 per annum, rising £5 per annum to a maximum of £60. The recommendation was adopted, and it was decided to advertise for a successor to the post which has to be filled owing to Miss Foyster's promotion.

### A NURSES' HOME FOR HONG KONG.

It has been decided by the subscribers to the Hong Kong Nursing Institution to secure a home for its nurses. This, however, would have been impossible at present, if Sir Paul Chater had not come forward and offered to collect or find himself the $10,000 required for the purpose. Hitherto, the two nurses have been housed at the Peak Hospital, and the scheme for the establishment of an independent home is to include provision for a matron. Dr. Stedman, who was previously opposed to the idea of a separate home, owing to the question of cost, admits that Sir Paul Chater, by his generosity, has removed the obstacle to its fulfilment.

### A WARNING TO MATRONS OF NURSING INSTITUTES.

A very hard case is reported to us from Bootle. The matron of the Institute for Trained Nurses in that town was last summer asked by the matron of another nursing home in Lancashire to send a nurse to a neighbouring isolation hospital. The request was made by telephone, and the next morning the nurse was sent. She remained for a week and the account was sent for £1 11s. 6d. and 7s. 6d. travelling expenses. Repeated applications for payment and the return of nurse's linen being sent in vain, a letter was addressed to the Secretary of the Isolation Hospital, who replied that the matron who supplied the nurse had already been paid. The solicitors to the matron of the Bootle Institute then naturally demanded payment from the person who telephoned for the nurse, but their communications have been ignored. So far no summons has been issued, and the matron at Bootle states that she wishes to avoid taking legal measures if possible. But having previously lost considerable sums of money through another matron pursuing a similar course, she asks us "if there is a remedy to prevent these losses." We are afraid that there is only one mode of absolute protection, namely, to refuse to send nurses to private venture homes unless the fees are paid in advance. This should not be necessary, but if matrons of nursing institutes cannot be depended upon to treat each other fairly, they ought not to have the chance of behaving in the manner described by our correspondent.

### INFIRMARY NURSES AND THEIR EXAMINATIONS.

An extremely satisfactory report has been presented to the Guardians of the City of London Union by Mr. Bruce Clark, who conducted the examination of the probationers. The successes amounted in each branch to 99 per cent., the best result obtained up to the present time. A motion was passed by the Guardians congratulating the medical superintendent and the matron in the outcome of their efforts in connection with the examination. The Hackney Board of Guardians have also received a satisfactory report from Dr. F. J. Smith concerning the result of the annual examination. Dr. Smith states that among the first year's nurses at least 50 per cent. showed more than ordinary ability and care, and he specially commended Nurses Morgan, Kitching, Dennis, and Mossman.

### DISTRICT NURSES AS AUXILIARY HEALTH INSPECTORS.

In the triennial report of the visiting nurses' work in connection with the Associated Charities of San Francisco, Miss Lucy Fisher gives a remarkable instance of the practical manner in which the district nurses of the city have developed into auxiliary health inspectors. A child was taken ill with smallpox, and the Board of Health fumigated the house in which the family resided. The nurses, however, did not feel satisfied, and reported to the Board that in that house the family had been afflicted during the year with measles, pneumonia, diphtheria, and small pox, that the city ordinance that only one wall paper at a time should be on the wall was being disobeyed to the extent that eleven layers were on one wall, and that the plumbing was far from the standard required by law. The result of the representation was that the landlord was compelled by the Board of Health to put in new plumbing and tear down the accumulation of wall papers with their concentrated germs. The badges worn by the district nurses of San Francisco give them the necessary authority to enter complaints directly against delinquent landlords, and papers to serve on them.

### A DIFFICULTY AT PENZANCE.

The Penzance Guardians have had a similar experience to that of the Board at Newport, Monmouthshire, which was reported a little time ago. They advertised for a nurse at a salary of £30, and received six applications, selecting two of the candidates. Both of the latter subsequently withdrew their applications. It has now been decided by the Penzance Board to offer a salary of £35, and an effort to fix the amount at £40 was only rejected by 15 votes to 13, after a discussion on the difficulty of getting certificated nurses to stay in the town. In such circumstances, the proposal of the minority seems to have been more likely to achieve the purpose in view.

### PROGRESS AT DERBY.

There are several interesting features in connection with the thirty-ninth annual meeting of the Derby and Derbyshire Nursing Association. The report stated that more work had been accomplished in private and district nursing than in any previous year of its history. The sum of £365 had been divided amongst the nurses as an extra bonus, and £467 added to the contingent fund for old and disabled nurses. The income of the association amounted to £5,363, and the expenditure to £4 531, the balance being £832. Nurses had been employed in 577 families and in 70 hospitals. The district superintendent and nurses paid in the borough of Derby 39,550 visits, and in districts 20,468, making a total of upwards of 60,000. During the meeting the mayor presented Nurse Edwards with a purse and £20 in recognition of her 20 years' service, and silver badges to Nurses Barker, Jackson, Edwards, Freeman, McCartney, Wainwright, and Routledge for 14 years' service.

### A SERIOUS DEFICIENCY AT WARWICK

It is rather surprising that the Mayor of Warwick in moving the adoption of the annual report of the Warwick District Nursing Association should have been compelled to draw attention to a serious deficiency. Lady Warwick, who is the president of the Association, has exerted herself so greatly on its behalf, that we should have thought the townspeople would have backed her up in a proper manner instead of subscribing only the small sum of £131 to meet an ordinary expenditure of £198. But for a donation from the Hunt Club, and the success which attended the charity ball, the reserve fund would have been exhausted. It is therefore clear that the ladies' committee and the collectors generally must set to work to put the Association on a sound financial basis.

### THE VICAR OF CROYDON AND THE NURSING MOVEMENT.

The appointment of the Rev. Leonard Hedley Burrows as Vicar of Croydon will deprive Godalming, a few weeks hence, of the services of two indefatigable friends of the nursing movement. Ever since the Meath Home of Comfort was opened in 1892, Mr. Burrows, as Vicar of Godalming, has been honorary chaplain and his wife honorary secretary. Mr. Burrows is also vice-president, and Mrs. Burrows a member of the house committee of the Victoria Nurses' Home at Godalming. In both these excellent institutions they will be greatly missed; but Croydon must, on the other hand, be congratulated on the fact that the successor to Bishop Pereira may be relied upon to prove a warm friend to all nurses and nursing associations in that great and still growing town.

### A GOOD BEGINNING AT SANDWICH.

The first annual meeting of the Sandwich District Nursing Association only attracted a small attendance to the Guildhall, but the report, which was adopted, was of a satisfactory character. The expenses were £74 3s. 5d. and the receipts £158 16s. 4d. The committee are therefore in the happy position of having a cash balance at the bank of £34 12s. 11d., and £50 on deposit. But we note that while the donations amounted to over £100, the annual subscriptions were only £45. As the nurse could not take up her duties until more than three months after her appointment, the amount for salary in the current year will be larger, and more subscriptions will probably be requisite.

### DISTRICT NURSING IN FLINT.

The little town of Flint appears to be fully alive to its obligations to the District Nursing Association. Last year an entertainment produced £26, and the third annual report, which was read and adopted at the yearly meeting of subscribers, shows that the receipts for the 12 months, including a balance brought forward, amounted to £142 as against payments £54. The question of providing a maternity nurse was discussed, and the state of the finance would justify the addition. As the district nurse attended 116 cases and paid 3,182 visits last year, her hands are clearly full.

### SHORT ITEMS.

Miss Isabel Jane Livingstone, the nurse who was charged with setting fire to the Booker Isolation Hospital, near Wycombe, was last week tried and acquitted at the Bucks Assizes.—The failure of Mary Anne O'Brien, of Yarrawanga, nurse, is announced in the Australian papers. Her liabilities are £74 10s. 7d. and her assets £15, a deficiency of £59 10s. 7d. The cause of the deficiency is attributed to a decline in the birth-rate.

### The Nursing Outlook.

"From magnanimity, all fear above;
From nobler recompense, above applause,
Which owes to man's short outlook all its charm."

### A QUESTION OF AGE.

There is a very general feeling, particularly amongst the public, that the minimum age at which a woman can be enrolled as a midwife, which stands at present at 21, should be raised.

No doubt the work of midwifery is not suitable for very young women. There is a good deal of responsibility, there is a good deal of dangerous knowledge connected with it; but there is something to be said on the other side. Firstly, the age is the minimum legal age, and it is left to the training schools to make it higher if they wish, each for their own pupils. Secondly, quite young lads are allowed to become medical students and gain dangerous knowledge, and, on the whole, the more youthful they are the less they are tempted by this knowledge. We are not anxious to see restrictions placed on women's work simply because the work is done by women. On the whole the female sex are purer minded than the male sex, simply because they are more carefully protected and trained in youth; and unless medical knowledge is to be forbidden to the student under 21, we do not wish it forbidden to the midwife under 21. Thirdly, a woman's working life is very short—nurses have found this out already. The nurse who enters at 25 and does her four years' training and only begins to earn full wage when nearly thirty, finds that she has only some twelve years of fully-efficient toil before her powers begin to fail; and during these twelve years she has to save probably for a lengthy old age, possibly some 30 years! Of course, the midwife should be more and more efficient as years go on and her experience deepens; just when the general nurse is finding the constant strain of passing from one serious private case to another is too much for her, the midwife is at her prime, and her working years should easily last up to 60.

So that the whole question comes to this: there is no necessity to alter the minimum legal age, but we sincerely hope that no midwifery training schools will give instruction to women under 21, except under the most exceptional circumstances. It is always possible that the young wife of some minister going as a missionary in far-off lands might wish to take her midwifery at an early age, and therefore it is well that rules should be as light as possible. It is easy enough for a training school to make a general rule, because the personal equation can be taken into consideration, and the matter brought to a vote before a committee if necessary; it is because the law is not easily altered, that it should always be as wide as possible.

The age of 21 is the legal age for enrolment under most of the nurse registration Bills now in force, but to be ready at this age means that a girl must commence her training at 17; and so far as we know no recognised training schools for nurses either in America, Australia, or Africa take probationers at this age. The best nurse-training schools in this country put the minimum age for entrance at 25; this is excellent from the point of view of the patients and the hospital authorities, and the independent nurse, but it is a serious inconvenience to the woman who has her living to earn. What is she to do from the time of leaving school until she is 25 years of age? And how is she to save for her old age in the few working years during which she is likely to find fully-paid employment? It is necessary to remember the business point of view at times, especially now that the profession is becoming more and more a mere business concern.

So long as we allow so short a period of training for midwifery as three months, it seems as though the best way out of the difficulty would be only to allow midwifery training to those who have already had general nursing training, and to allow probationers to enter the general hospitals at a minimum age of 21. But this should be a question for each training school: not a question for the House of Commons. Laws are bound to press hardly on exceptional cases, and the fewer laws we have the better under all circumstances. Therefore it is best to hold firmly to our conviction that no woman under 25 should be trained as a midwife, but meanwhile to allow the minimum legal age to stand until we have evidence that its modest limit has been abused.

It is greatly to be regretted that it is usual amongst women to pay so much importance to the question of age; there are hundreds of women who give up working, for instance, just when at their best because of this fancy that a woman grows old sooner than a man. The far truer and better saying is that a woman is as old as she feels, and we know of at least one lady of 70 who is still in active work as directress of an institution; had she retired at 50, as was suggested to her by several of her friends, she would have missed bringing to perfection a fine piece of work, and might have marred her early record by leaving a memory of a fretful and trivial old age. In the same way, seeing how short life is at the longest, it seems a mistake to keep girls ignorant and useless till they are well on in their twenties. The eagerness for work is strongest in our teens, and nature certainly meant us to develop and to marry far earlier than is possible for the majority under our present civilisation. Let us leave all age-limits as wide as possible.

### The Modern Nursing of Consumption.

By Dr. Jane H. Walker, Physician to the New Hospital for Women, London, and Medical Superintendent of the East Anglian Sanatorium, Nayland, Suffolk.

### LECTURE VIII.

### Special Points in Nursing.

*Diets.*—In the nursing of private cases the nurse may be called upon to suggest suitable diets for the patient, especially in cases where there is sickness or diarrhœa. A milk diet is often airily recommended by the medical attendant, and it will devolve upon the nurse to make this diet as little irksome as possible to the patient, such things as Benger's food, Allen and Hanbury's malted foods, Mellin's food, Horlick's Malted milk, or Robb's biscuits soaked in milk will suggest themselves to any nurse's mind. She should see that all milk puddings are really well made, that arrowroot is not lumpy, and ring the changes upon all the various flavourings to make the food as palatable and little monotonous as she can. In the place of ordinary milk, especially if the large quantities required be objected to, evaporated milk may be used. It is made by boiling milk in a double saucepan until reduced to half its original bulk.

*Sickness.* If a patient be frequently sick the diet should be changed for a day or two, and an entirely milk diet be tried, the milk being mixed with one fourth part of barley, lime, or soda water. Should the milk diet not agree a meat one may be better. Minced meat, lightly cooked, raw meat sandwiches, with toast and meat juice will often agree well.

The best way of making raw meat juice is to get good beef steak, free from fat, cut it into small pieces, and squeeze each piece in a wooden lemon squeezer until all the juice is expressed. To a wineglassful of the juice so obtained add an equal part of Bovril, Liebig, or ordinary beef tea flavoured with celery-salt, or herbs. Give a dessertspoonful or more whenever ordered. The most fastidious invalid will never know that he is not taking ordinary beef tea. The beef tea added to the juice may be slightly warmed, but must not be hot or the juice will coagulate.

Of meat extracts Brand's jelly and Valentine's beef juice are the best. Most patent, and for that matter home made, beef teas contain no nutriment, and are useful only as being more or less palatable and rather stimulating.

Some patients who suffer from sickness or dyspepsia do well if they take nothing but meat and toast in the middle of the day, and vegetables and pudding in the evening. This plan of giving the bulk of the proteid and the carbohydrate food separately is specially useful in cases of flatulent dyspepsia. Five grains of bicarbonate of soda with half a teaspoonful of sal volatile half an hour before meals often aids in keeping off sickness and indigestion.

If for any reason a patient be put upon a special diet for a time every effort should be made to get him back to his ordinary meals as soon as possible.

*Diarrhœa.*—In the later stages of phthisis uncontrollable diarrhœa generally means tubercular affection of the intestines, and is a very serious condition. The doctor will give the appropriate remedies, the diet will also probably be entirely regulated by his orders. Milk, arrowroot, or a meat diet, with stimulants such as brandy, whisky, or champagne are most likely to be ordered, and these dietaries may have to be perpetually varied. In some cases of sickness and diarrhœa, when nothing else will agree, pancreatised arrowroot will be found most useful. It is made as follows:—

To one pint of boiling water arrowroot add one pint of cold milk, two teaspoonsful of liq. pancreaticus, and 40 grains of bicarbonate of soda. Stand for 20 minutes in a warm place near the fire, boil for 10 minutes, strain. It can then be given in any suitable quantities. The chief objection to this arrowroot is that it tastes nasty and flat, and requires considerable care in flavouring to make it palatable. Occasional attacks of slight diarrhœa are best treated, in the absence of the doctor, by a dose of a quarter of an ounce of castor oil, in the same quantity of whisky, to which, if the doctor permit, five minims of tincture of opium can be added. At the same time the diet for a day or two should consist of milk and milk puddings only, with toast or a crisp rusk. Always remember that when a patient is on a milk diet, his tongue tends to get thickly coated with fur, and his mouth to taste disagreeable, and that anything like a small piece of toast or a biscuit, slowly masticated, will clean the tongue and make him feel much more comfortable.

*Pain.*—The most common sites of pain are either at the bases of the lungs behind, or in the axillary line, or just below the clavicles in front. Pain is frequently only caused by intercostal neuralgia, but may be a sign of pleurisy. In any case, if severe, the doctor should be sent for. In the meantime the nurse should apply hot fomentations. Flannel or spongiopiline wrung out in hot water and sprinkled with tincture of opium are generally very soothing. The patient should be kept quiet, and if for any cause the doctor cannot come till the next day, relief may generally be obtained by strapping the affected part. Ordinary coarse hospital strapping in strips about one and a-half inches wide should be laid on, the edges of each successive strip overlapping about half an inch. This, by keeping the part at rest, will relieve pain, whether caused by neuralgia or by the tension of pleural adhesions.

*Hæmorrhage.*—In the early slight attacks of hæmorrhage nothing beyond keeping the patient quiet for a day or two need be done. Small streaks of blood in the sputum are no indication for altering diet, or for complete rest in bed, but much exercise should not be taken.

The late, large, and often fatal hæmorrhages must be dealt with immediately if life is to be saved. The handiest thing a nurse can use is turpentine. A few drops sprinkled on a piece of lint or cotton wool and held to the mouth and nostrils will often prove efficient. In the case of patients very ill and with large cavities, likely at any moment to lead to hæmorrhage, the nurse should always have turpentine in the house, if not in the room, at least where she can lay her hand on it at a moment's notice.

For some days, or even weeks, after a bad hæmorrhage, the patient must be kept absolutely quiet, and not allowed to do anything whatever for himself. A bed-pan should be used, no visitors allowed, and he must even in many cases be fed in bed by the nurse. A pulse of high tension, especially if the tension suddenly become higher, should make the nurse look out for hæmorrhage in a case known to have a cavity. Constipation should be guarded against; calomel, one grain at night, followed by a dose of salts in the morning, will generally reduce the pulse tension, or if there have not been any hæmorrhage an enema may be given. It is generally not wise to give an enema just after a hæmorrhage, as it agitates the patient too much. Of enemata we prefer glycerine, or small oil ones as being less disagreeable to the patient than large ones of soap and water, and they are equally efficient if given high up with a small-sized rectal tube. If a soap and water enema be given its size may be reduced while its efficacy is maintained, if an ounce of castor oil be added.

*Dyspnœa* (Breathlessness).—This is at times a most distressing symptom. The patient should be well propped up in bed; sometimes a firm bandage round the lower ribs gives relief. Probably various drugs will be ordered by the doctor, which, in a private case, the nurse will have to use some discretion about giving, as alarming attacks of dyspnœa may often occur in the absence of the medical attendant.

*Kidney Affection.*—In the later stages of phthisis kidney troubles may supervene. The diet and treatment must depend upon the doctor. The nurse should remember that cases apparently hopeless may get very much better and live for months or even years, and that the occurrence of kidney mischief, though making the outlook extremely grave, need not be at any rate an immediately or rapidly fatal symptom.

*Bronchitis.*—This is often indicated by much cough. If the cough persist the doctor should be sent for, as a nurse cannot tell the cause of the cough.

*Nose and Throat.*—Many patients whose throats are not tubercular suffer from pharyngitis and unhealthy tonsils, and frequently have much sticky discharge at the back of the nose and throat, especially in the mornings. For this discharge the best remedy is sniffing an alkaline solution up the nose night and morning. A solution of the strength of half a teaspoonful of common salt with half a teaspoonful of bicarbonate of soda to a tumbler of warm water should be used. The lotion may either be sniffed up from the palm of the hand or from a small piece of sponge, allowed to run down the back of the throat and then spit out. A little practice is required to do this with ease, but it is really not difficult to get into the way of sniffing up four or five ounces in as many minutes. Painting the pharynx with glycerine or boracic acid, or some preparation containing iodine, as ordered by the doctor, generally gives relief in cases of dryness of the back of the throat.

If the larynx be affected with tuberculosis, there is generally more or less complete loss of voice, often pain, especially when swallowing food, and at times much irritating and distressing cough. Various insufflations will probably be ordered by the doctor. Patients can be taught to insufflate their own throats. If the nurse has to do it she should wrap a piece of cloth (thickish diaper cloth is the best) round the tip of the tongue. Then, grasping the tongue firmly with the finger and thumb of her left hand, she pulls the tongue well forward, and introduces the end of the insufflator into the mouth, keeping the end of the nozzle well against the back of the tongue with the extreme end pointing down to the larynx, she should then give the rubber ball a sharp squeeze, and the powder ought to get into the larynx. If the end of the nozzle be not kept depressed, or if it be too much against the back of the throat, the powder will go down the œsophagus. The same precautions must be followed in using a laryngeal spray.

When there is much difficulty and pain in swallowing morphia or cocaine insufflations are often ordered, and in these cases special care must be taken that the preparation goes only into the larynx.

Menthol lozenges often give relief in cases of hoarseness and dry throat; they can safely be given to anyone. Morphia lozenges are also of use in cases with irritating dry cough, but their use should be regulated by the doctor's orders.

*Night Sweats.*—These are as a rule unknown if the patient be kept really on the open-air treatment, but may occur in acute cases, or in cases in the last stages of the disease. The patient should be sponged over with lukewarm water with a little boracic acid or vinegar in it; this will often be grateful to the patient, though it has no effect upon the sweats. Many phthisis patients always perspire unduly though there are no night sweats. Sponging the body over after bathing, with weak boracic lotion, or dusting, and especially dusting the axillæ, with equal parts of starch and boracic acid powder may be useful.

*Pyrexia.*—If the temperature be above 101° F. rest is the most important point in the treatment. If over 105° F. sponging may be tried. Cold baths, rubbing with ice, or an ice bag to the head are only required in exceptional cases of acute tuberculosis. It is generally better if cold baths have to be used, to put the patient into one at 80° F. and gradually lower the temperature. Some stimulant, such as brandy or sal volatile, should be at hand in case the patient is collapsed after the bath. In ordinary cases of phthisis the daily rise of temperature even in bad cases does not exceed 103° to 104° F., and beyond the most absolute rest no special treatment is required. Patients with a temperature every evening of 103° F. can take ordinary diet as a rule, though the amount may have to be reduced in the evenings, or the meal time altered so that it may not coincide with the maximum temperature.

### How to Become a Midwife: The Rules of the new Act Explained.

By E. L. C. Appel, M.B., B.S., B.Sc.(Lond.).

*Continued from page 164.*

Third Way.—*For women who have obtained a certificate in Midwifery from any institution or examining body (approved by the Central Midwives Board) other than* the Royal College of Physicians of Ireland, the Obstetrical Society of London, the Coombe Lying-in-Hospital and Guinness's Dispensary, or the Rotunda Hospital for the relief of the Poor Lying-in-Women of Dublin (see Appendix, p. 29).

If you have a certificate in Midwifery, but did not get it from any one of the four above-named bodies:—

1. Write for Midwife Form 7.[5]

*Form* 7 is:—I hereby testify that before a certificate was granted to..........................by the institution or examining body of which I am at present the accredited or recognised representative..........................had received a proper course of instruction and training (including personal attendance under competent supervision upon at least twenty cases during and after labour), and had passed an examination in midwifery and the duties of a midwife.

I further testify that she is at the present time a fit and proper person to be admitted to the Midwives Roll.

Dated this...day of.........19....

Name......................................

* Chairman of the Board, or Committee, or Senior Medical Officer of the ...............

..................................................

Signature of applicant..........................

* Strike out such words as do not apply to the person signing.

Form 7 must be filled in by a person in authority at the institution from which you obtained your certificate in midwifery, and you must write your name after the words "Signature of applicant." You may be required to furnish other documents or particulars to enable the Central Midwives Board to decide whether the application can be granted.

[5] These articles are published in book form by the Scientific Press, Ltd., 28 and 29 Southampton Street, Strand, W.C., at 1s., and 1s. 1d. post free. All midwife forms, price 1d. each, postage ½d. extra, can also be obtained from the Scientific Press.

### HOW TO BECOME A MIDWIFE—*Continued.*

2. Send Form 7, properly filled in and signed, together with your certificate in midwifery and the fee of ten shillings, to the Secretary of the Central Midwives Board, 6 Suffolk Street, Pall Mall, London, S.W.

3. In return, you will receive from the Central Midwives Board a certificate (*Form* 10) as follows:—

Central Midwives Board.

(2 Edw. 7. c. 17.)

*No.* ......... *Date* ....................

We hereby certify that ............................ is entitled by law to practise as a midwife in accordance with the provisions of the Midwives Act, 1902, and subject to the rules and regulations laid down in pursuance thereof, by virtue of holding a certificate in midwifery from .......................

............................ } Members of the Board.
............................

...................., Secretary.

This makes you a certified midwife.

*Certification after March* 31, 1905.

After March 31, 1905, every woman who is not already certified under the Midwives Act, and who wishes to become a midwife, will be required

1. To produce three certificates showing she has undergone the full course of training for midwives, prescribed under Section C of the rules of the Central Midwives Board (see Appendix, p. 28).

2. To produce a certificate of birth, showing that she is not under 21 years of age.

3. To produce a certificate of good moral character.

4. To pass the examination of the Central Midwives Board.

The prescribed course of training requires

I. *Attendance on Cases.*—The woman must, under supervision satisfactory to the Central Midwives Board, attend and watch the progress of not fewer than 20 labours, making abdominal and vaginal examinations during the course of l.bour, and personally delivering the patient.

II. *Attendance during the Lying-in Period.*—She must, to the satisfaction of the person certifying, nurse 20 lying-in women during the 10 days following labour.

III. *A Course of Instruction.*—She must, for a period of not less than three months, attend a course of instruction in the following subjects, given by a doctor recognised by the Central Midwives Board as a teacher:—

(*a*) The elementary anatomy of the female pelvis and generative organs.

(*b*) Pregnancy and its principal complications, including abortion.

(*c*) The symptoms, mechanism, course and management of natural labour.

(*d*) The signs that a labour is abnormal.

(*e*) Hæmorrhage: its varieties and the treatment of each.

(*f*) Antiseptics in midwifery and the way to prepare and use them.

(*g*) The management of the puerperal patient, including the use of the clinical thermometer and of the catheter.

(*h*) The management (including the feeding) of infants, and the signs of the important diseases which may develop during the first ten days.

(*i*) The duties of the midwife as described in the regulations.

(*j*) Obstetric emergencies, and how the midwife should deal with them until the arrival of a doctor. This will include some knowledge of the drugs commonly needed in such cases, and of the mode of their administration.

(*k*) Puerperal fever, its nature, causes, and symptoms. The elements of house sanitation. The disinfecting of person, clothing, and appliances.

Her *certificate of character* and her three certificates of having undergone the above course of training must be on schedules, *i.e.* on Forms 1, 3, 4, 5.[6]

*Form* 1 (Certificate of Good Moral Character) is:—

I certify that I have been personally acquainted with ............................ for a period of ........... years, and that she is trustworthy, sober, and of good moral character.

Dated this ........... day of ..............., 19...
Name ..........................................................
Address ......................................................
Position and authority for signing ..........................
Signature of applicant.........................................

Form 1 must be signed by a person who has known the candidate for at least 12 months, and the candidate must write her name after the words "Signature of applicant."

*Form* 3 (Certificate of Attendance on Cases) is:—

I certify that ...................................... (to whom this certificate refers) has, under my supervision, attended and watched the progress of not fewer than 20 labours, making abdominal and vaginal examinations during the course of labour, and personally delivering the patient.

Dated this ........... day of..............., 19...
Name ..........................................................
Address ......................................................
Position and authority for signing.............................
Signature of applicant.........................................

*Form* 4 (Certificate of Attendance during the Lying-in Period) is:—

I certify that ................................ (to whom this certificate refers) has, to my satisfaction, nursed 20 lying-in women during the 10 days following labour.

Dated this ........... day of ..............., 19...
Name ..........................................................
Address ......................................................
Position and authority for signing.............................
Signature of applicant.........................................

Forms 3 and 4 must be filled in "either by a registered medical practitioner or by the chief midwife, or, in absence of such an officer, by the matron of an institution recognised by the Central Midwives Board, or, in the case of a Poor-law institution, by the matron, being a Midwife certified under the Midwives Act, or a superintendent nurse, certified in like manner and appointed under the Nursing in Work-houses Order 1897 and attached to such an institution, or by a midwife certified under the Midwives Act and approved by the Central Midwives Board for the purpose."

The candidate must write her name after the words "Signature of applicant."

*Form* 5 (Certificate of having Attended a Course of Instruction) is:—

I certify that ...................................... (to whom this certificate refers) has attended, to my satisfaction, a course

[6] See Note 5, p. 179.

of instruction given by myself on the subjects enumerated in the regulations.

Dated this...day of........., 19...

Name ..................................................

Address ..................................................

Professional qualifications ..............................

Position and authority for signing ........................

Signature of applicant......................................

Form 5 must be filled in by a registered medical practitioner recognised by the Central Midwives Board as a teacher, and the candidate must write her name after the words "Signature of applicant."

*The Examination*[7]—The candidate will be examined in the subjects of the course of instruction, named above (see p. 11). The examination will be partly oral and practical, and partly written.

The candidate must, at least three weeks before the date fixed for the examination to begin, send notice to the Secretary of the Central Midwives Board, stating that she intends to present herself for examination, and enclosing her five certificates (viz., Forms 1, 3, 4, 5, and her certificate of birth), together with the fee of one guinea.[8]

The candidate must satisfy the Central Midwives Board that she has reached a sufficient standard of general education. If during the examination she shows a want of acquaintance with the ordinary subjects of elementary education she may be rejected on that ground alone.

If the candidate successfully passes the examination she will receive from the Central Midwives Board a certificate (schedule, Form 2) which is as follows:—

Central Midwives Board.
(2 Edw. 7. c. 17.)

*No.* ...... Date ..............

We hereby certify that ................................ having passed the examination of the Central Midwives Board, and having otherwise complied with the rules and regulations laid down in pursuance of the Midwives Act, 1902, is entitled by law to practise as a midwife in accordance with the provisions of the said Act and subject to the said rules and regulations.

........................ } Members of
........................ } the Board.

............................ Secretary.

This makes the candidate a certified midwife.

*The Roll of Midwives.*

The name of every certified midwife will be entered on the Roll of Midwives by the Secretary of the Central Midwives Board. The names of all women admitted to the Roll of Midwives will be printed annually in alphabetical order. A current copy of the Roll of Midwives printed by the authority of the Board or signed by the Secretary to the Board will be evidence in all Courts that the women therein specified are certified under the Midwives Act.

A current copy of the Roll of Midwives will be kept by every local supervising authority in England and Wales, and will be accessible at all reasonable times for public inspection.

The Central Midwives Board is empowered by law to remove from the Roll of Midwives the name of any midwife who disobeys the rules and regulations or is guilty of other misconduct, and may also cancel her certificate.

7 Due notice will be given of the examinations to be held under the Act.

8 For a candidate who presented herself for examination on a former occasion and failed to pass, the fee is 15s.

*(To be continued.)*

### Nursing Questions at the International Congress of Women in Berlin.

BY AN ENGLISH MATRON.

Among the countless beautiful buildings which Berlin, the City of Palaces, contains, by no means the least magnificent is that where the Congress of 1904—the Philharmonic, in Bernburger Strasse—has been held. It has been *en fête*, its cool entrance courtyard a fairyland of graceful birches and festoons of greenery. It is admirably adapted for the purpose, though ordinarily it is devoted to music, for it contains several handsome halls where meetings can be held, and it has besides committee rooms, a post office, a Press room, ticket and inquiry offices, and prettily arranged reception rooms.

If there was a fault to find with the Congress, it was that so many interesting papers were being read at the same time, making it impossible to be present at all of them.

Speaking generally, those sections that dealt with Poor Law, sick nursing, and the position of wage-earning women, were more directly of interest to nurses, and many excellent papers were read on those subjects, irrespective of those given at the session on Thursday especially devoted to nursing.

### Nursing by Nuns in Austria.

Thus, Frau Hertha von Sprunge, speaking on the subject of Poor Law in Austria and dealing also with hospitals and nursing, made some startling statements as to the death-rate amongst boarded out children. She also alluded to the alarming mortality amongst the nuns engaged in sick nursing, which she placed as high as 50 per cent. among those under 30, attributing it to overwork and lack of proper organisation. The lay workers, she stated, are so badly paid and so inferior, that it is absolutely necessary for the nuns to work far beyond their strength to insure any kind of proper attention to the sick. She severely criticised the short training of midwives, who, as throughout the Continent, are far more generally employed amongst the better classes than in England. The religious question, she said, seems throughout to complicate matters in Austria.

### The Women's Association in Baden.

Further papers in the same section dealt with the almost perfect organisation of the Women's Association in Baden, founded 53 years ago, during the war in Italy, by the then Grand Duchess. This embraces every form of charitable work, with a section for sick nursing, which, amongst other duties, provides district nurses throughout the Duchy, even in remote country districts, thus anticipating our Queen's Jubilee nurses. It was impossible not to admire the able manner in which Frau Alice Bensheimer explained how the branches of the Women's Association spreads like a network throughout the Duchy, practically absorbing all charitable work, and entirely obviating that wasteful overlapping which is such a prominent feature of English charitable organisations.

An excellent paper was read by Miss Thomson of Canada on district nursing there, whilst a lady doctor, Mrs. Alvida Harbon-Hoff, from Denmark, gave some interesting views as to the "struggle with tubercular disease in childhood." Altogether thirteen papers were read in that day in that section, every one of which was of more or less interest to nurses. The three languages used were German, French, and English.

The halls in which Frau Stütt, Fraulein Lange, and other popular speakers were speaking were always packed, and, whilst the audience applauded liberally they were not slow to express their disapproval of unpopular doctrines. Great efforts were made by the ladies who acted as stewards to keep order in the halls during the speeches, but it was very difficult to prevent the audience from wandering from hall to hall to listen to different speeches in different sections, so that there was, except during speeches of absorbing interest, a continual subdued noise. Otherwise the whole organisation was most excellent, and a marked feature of the Congress was the courtesy and readiness of address of the chairwomen.

The Nursing Session.

The Thursday session in the Second Section (Women's Professions and Occupations) was held in the great hall, and was devoted entirely to the question of nursing. The Chairwoman, Frau Elsbeth Kreuznach—who opened the sitting with a short but able address—was followed by Mrs. Bedford Fenwick with a paper on nursing from an educational, industrial, and social aspect. After stating the necessity for a thorough and full education for nurses, Mrs. Fenwick dwelt upon the need of a proper organisation of the profession, of the need of cultured women. The better educated the nurse, the more likely to do credit to her profession. The modern nurse should be trained to meet scientific requirements. Mrs. Fenwick strongly advocated the preliminary and post-graduate courses and emphasised the cost of a modern nurse's training, suggesting that it should be made a matter of State aid, as, like preventive medicine, good nursing was of the greatest service to the nation by securing the greatest efficiency in the nursing of the sick and the care of children. Part, too, of the cost of training should be borne by the student. Mrs. Fenwick, in conclusion, pleaded for the need of a proper organisation of the profession, for State registration and State examination. She spoke clearly in English.

She was followed by Miss Dock on the organisation of nursing in America, and by Sister Agnes Karll, of Berlin, who gave a most interesting and popular appeal for the "Free Sisters," as opposed to those who work in the Deaconesses institutes—the most usual and approved form of association in Germany—and for an excellent course of training to be followed by State recognition. This speaker was followed by eight others, English, American, Italian, and French, almost all of whom treated the subject mainly from an educational and industrial point of view.

Many of the speakers gave eloquent testimony to the work accomplished by the deaconesses and the Red Cross society in Germany, but others spoke of the desire of some German nurses to escape from the never-ending personal control which enfolds a woman from the moment she enters such an institution. Professor Zimmer, a well-known authority on nursing matters, and Dr. Israel, both spoke in this section, and the desire for some State recognition of the status of a trained nurse in Germany was practically universal. Various accounts were given of the education and work of nurses throughout Europe. The discussion was animated and well sustained. There was an attendance of about eighty English and American nurses, besides, of course, Germans, but it was regrettable that so few deaconesses were present.

Hospitalities to Nurses.

Any account of the Congress would be incomplete which did not mention the lavish hospitality of the Germans and the festivities which were organised by them in honour of the Congress members. Whilst every morning was passed in debate, every afternoon was spent in attending receptions in private houses and clubs, garden parties, water parties, and exhibitions to which invitations were literally showered on the members.

### International Council of Nurses.

The quinquennial meeting of the International Council of Nurses was held in Berlin on Friday last at the Victoria Lyceum, Potsdamer Strasse, Mrs. Bedford Fenwick in the chair. There was a good attendance, among those present being Miss Isla Stewart, Miss Rogers, Miss Pell Smith, Miss Jones, Miss Mollett, Lady Hermione Blackwood, Miss Breay (England), Miss Dock, Miss Goodrich, Miss Thornton, Miss B n ield (America), Fräulein Agnes Karll (Germany), and many others.

Considerable business was accomplished, including the passing of reports and the accounts concerning the financial position of the Council, and details of its progress since the last meeting. The following officers were also nominated for the next quinquennial period:—Miss McGay (Sydney), president; Miss Dock, secretary; and Miss Breay, treasurer.

The next question that came under consideration was that of the Affiliation of National Councils of Nurses, which was put before the meeting by Mrs. Bedford Fenwick (London), Miss Thornton (America), Miss Stewart (England), and Sister Agnes Karll (Germany). A proposal that an invitation be sent to the Federations of American and German nurses to affiliate into the International Council of Nurses was passed unanimously.

After lunch (for which there was an adjournment of two hours) digests of the reports on Government Services (naval and military) were read, it being pointed out that England and America are the only two countries with organised naval and military services.

The progress of the State registration of nurses, and the systematising of the education of nurses was next considered.

Miss Dock described the system and working of the registration by the State in America; Lady Hermione Blackwood read a report explaining the Act in force in New Zealand, and the system of qualifying examinations; Miss Dock also read a paper describing the progress made in Australasie, and Miss Mollett another report throwing light upon the nursing question in South Africa. Miss Goodrich then read a paper by Miss Nutting, dealing with the question of the adequate education of nurses. Stress was laid upon the importance of adequate preliminary education; preliminary technical education under qualified instructors; and clinical education.

A resolution moved by Mrs. Bedford Fenwick in favour of these points and of State registration and certification was unanimously passed by the meeting, which closed with a vote of thanks to Mrs. Bedford Fenwick.

### West Ham Hospital.

THE NEEDS OF THE NURSING STAFF.

A large number of residents in West Ham and the neighbourhood availed themselves of the invitation of the Committee and staff of the West Ham and East London Hospital to the third annual "At Home" on Saturday afternoon last. The guests were received by the Mayor and Mayoress, Mr. and Mrs. McDowall, and the matron, Miss Ough, flowers and music in the entrance hall adding to the effect of the welcome. Refreshments were dispensed in the exercising yard—at present the only available open space—and here were more flowers, on a stall and in trays carried by the nurses, while the band of the K Division Metropolitan Police played in the adjoining garden of the Girls' High School.

The main feature of the afternoon's proceedings was the throwing open for the occasion of the drill hall attached to the school, since it has been decided to purchase the school and grounds—and to convert the class-rooms into wards, receiving-rooms, out-patient department, etc., the drill hall itself being utilised for recreation in wet weather.

The absolute necessity for extension, in view of the rapidly increasing strain on the available space, was emphasised by the Mayor, who in a brief speech appealed to the inhabitants of the borough to rally round the hospital at this unique opportunity, so as to enable the scheme to be carried out with benefit to all concerned. He stigmatised the present accommodation—viz., 55 beds for a population of something like 500,000—as an absurdity, and added that more open space, as well as a new operating theatre, and medical and ophthalmic wards, were urgently required.

Dr. P. J. S. Nicholl, in appealing for the £15,000 to £20,000 required to carry out the scheme, remarked that the hospital was in many respects quite out of date, and that it was peculiarly deficient in the provision of adequate cubic space per patient.

Although not publicly alluded to by either of the speakers, adequate provision for an augmented nursing staff will, of course, have to be made, and the housing of the nurses is rendered the more important since, at present, the night nurses' rooms are beneath the kitchens. A collection was made during the afternoon by some of the nurses, who were distinguished by a broad gold band, with the words "Extension Fund," worn across the shoulder.

An excellent series of entertainments was given in the drill hall, including concerts, monologues, and a comedietta entitled "Cut Off with a Shilling," and there was an exhibition of the $x$-rays in the dispensing-room. Several ladies and gentlemen also sang in the wards, and the patients were further entertained with a phonograph. Each guest, on leaving, was presented by the matron with a current weekly, containing an illustrated account of the hospital and a detailed description of the proposed extension. Although the hospital is gaining a measure of recognition from outside —it has recently received a large grant from King Edward's Hospital Fund—the burden of support undoubtedly rests with the inhabitants of the neighbourhood, and the Mayor's promise to do his best to secure a grant from the Corporation was warmly received.

### Everybody's Opinion.

[Correspondence on all subjects is invited, but we cannot in any way be responsible for the opinions expressed by our correspondents. No communication can be entertained if the name and address of the correspondent are not given as a guarantee of good faith, but not necessarily for publication. All correspondents should write on one side of the paper only.]

### ST. JOHN'S NURSES.

Mr. E. R. Frere, Secretary, St. John's House, 7 and 8 Norfolk Street, Strand, W.C., writes:—My attention has been drawn to an interview published in your issue of June 18th, in which the history of this institution is claimed by the Community of St. John the Divine. The facts are these: In 1883, owing to a question between Council and Sisters, the sisters then on the active list, and an inconsiderable number of the nurses, sent in their resignations, which were accepted. There was, however, no break in the continuity of any one of our various works. The nursing staff was practically intact, and the Council, which included some of the original founders, remained in possession of the premises and assets, and, with the help of some former sisters who returned to us, we were enabled to carry on all our current works, including the nursing of King's College Hospital (until 1885) and Charing Cross Hospital (until 1889). St. John's House was projected by Dr. Todd in 1847, and first took shape under Miss Frere in Fitzroy Square in 1848. It is one of the oldest nursing institutions in England, and our members naturally feel some indignation when they see their history from 1848 appropriated by a community which only came into existence in 1883. This community did not take away with them any one of the works which were being carried out by St. John's House, and the good work which they are now doing is all new since they separated from this institution in 1883. As you have published your interview with the Sister Superior of St. John the Divine, I shall feel much obliged if you will insert this letter in your next issue as a contradiction to the statements, which amount to an assertion that St. John the Divine is the original institution."

### THE THREE GRADES IN WILTSHIRE.

Miss Amy Hughes writes: Without further reference to the Wiltshire Nursing Scheme, beyond stating that the aim of the Association in providing three grades of nurses is to meet all needs of district nursing in the county, I wish to endorse the opinion of "Inquirer" as to the inadequate salaries of fully-trained nurses in many district posts. Education as to the "market value" of trained nursing services, so equally essential for district as for hospital or private work, is much needed. Naturally promoters of nursing associations seek to carry out their schemes as economically as possible. The real solution of the problem lies with nurses themselves. The Queen Victoria's Jubilee Institute for Nurses stipulates for a minimum salary of £30 per annum, with allowances for uniform, board, lodging and laundry expenses. These nurses, in addition to full hospital training, have had special training in district nursing, and frequently hold a midwifery certificate. Constantly, however, one hears the statement that "Queen's Nurses are too expensive." Yet such remuneration is only the average annual income that can be obtained by nurses in the open market. If fully-trained nurses would refuse to accept salaries of less value than their training warrants, and so cease to undersell their fellow-workers, the many cases similar to the one quoted by "Inquirer" would become impossible. Naturally, so long as committees can advertise and secure nurses holding a three-years' certificate from a leading London hospital, and the L.O.S certificate for £60 inclusive per annum, and a bicycle, so long will such associations refuse to give a fair remuneration to their nurses, and encourage others to do the same. If nurses realise more fully the grave responsibilities incurred in district work they will cease to accept posts where the hire is unworthy of the labourer.

### THE PREVENTION OF BED-SORES.

"A St. John Nurse" writes: I have been much interested in the answers to the Examination Questions respecting "Bed-sores, their Cure, etc." I have had a patient in bed now for 18 months suffering from renal abscess. She has tubes over each loin, and is in a continual state of being saturated with pus. Pus is also passed in her urine, and often she has incontinence. So far she has had no bed-sore, nor any approach to one, though she is in an almost helpless condition. I have kept her scrupulously clean, frequently dressing the wounds on each loin. I take out the tubes, and wash freely with good soap all over the back, hips, etc., then rub methylated spirit well into the skin, first covering the wounds with a piece of old linen. Then I make an ointment of equal parts of vaseline and barff boro glyceride, and rub this into the skin all round the wound, and anywhere where the urine or pus is likely to reach. I have tried this before for patients, and have always seen the same good results. As none of your correspondents seem to have mentioned the use of barff boro, it may interest them to hear of it. It can be obtained of most chemists I also have a night light called the "Carbona." It is a small tin box filled with asbestos, and a thread of the asbestos is pulled through a small hole with an ordinary pin. I place the tin in a potted meat jar of paraffin oil for a few seconds, wipe it quite dry, and it is ready for use. It cost 3½d. a year ago, and it costs about 1d. a week for oil. The night light is as good as ever and has been in constant use for a year. It is such a saving to patients where illness is such an expense, and all these small things make a great difference. I got the light at an oil-shop.

PRIVATE NURSES AND FANCY WORK.

"Justice" writes: I am somewhat surprised at a member of our profession making such an unjust criticism as "the patient paid for the nurse's services and time and did not pay for the nurse to do her own fancy work when on duty?" Surely no matron with personal experience of how hard a private nurse's life is would ever have coincided with such a statement! Would she not reply, "Nurse was only sent to you to nurse faithfully, to the best of her ability, and take the responsibility of your invalid in the doctor's absence; carry out his orders, and attend to whatever came in her way for the invalid's wellbeing, mentally and physically." When money is scarce, it is usually only in heavy cases that a nurse is engaged, and when plentiful the family would engage sufficient domestics and otherwise for their household requirements, sewing included. Even if they care to engage a nurse for a trivial case it is their own choice, and surely nurse has not that to consider? I think we nurses all prefer a case where hard nursing is required. Personally, in four years' private work, I have had a very happy, though hard time—light cases have been short and very seldom. Fancy work has been practically impossible. In the greater part of my time it has been a relief to me when I had time to do personal mending and necessary needlework in plain sewing. Fortunately the people I have worked amongst have been exceedingly kind to me, and I am sure that any reasonable doctor or matron who received from patients, or friends, such a ridiculous complaint or criticism as "Matron" has made would certainly ask "Did nurse do her duty by her patient?' and if, as in the case matron speaks of, the reply was in the affirmative, I think nurse's reputation would not suffer, and the people would probably be reminded they were unreasonable. I hope that in an emergency, whatever house a nurse was in, she would do her best to help, but to look on family darning as part of her lot I hope never. Quite willingly we give up many pleasures, much of our sleep, the best years of our life, and our best energies, possibly somewhat selfishly. We love our profession. On the other hand all humanity loves gratitude. I have been too busy to follow up carefully State registration, but as I am shortly giving up private work for hospital again, I hope to live once more under hospital regulations, when regular rest, daily off duty, and days off helped one through the hard days. Private families are apt to forget that a serious illness with heavy nursing, is not in our experience, as in theirs, an occasional matter—but our very life, and when we finish with this particular invalid, we invariably go off with little rest or none to another case. So it goes on continuously if one is on a large staff, with a good connection, and with a reliable set of nurses. Matron suggested a report when a nurse's personal habits are not nice. The remedy surely is a request for the nurse to act differently or send her back to institute, and thus weed from the profession unsatisfactory persons.

### Novelties for Nurses.

By our Shopping Correspondent.

### A BRITISH EAU DE COLOGNE.

Many of our readers know to what perfection the manufacture of Eau de Cologne has been brought in the Channel Islands; but there are many to whom Luce's Jersey Eau de Cologne would be a revelation. This delightful perfume ranks with the very best of its kind, and in my opinion there is only one brand hailing from Cologne itself which can be regarded as a rival. The Jersey scent has the advantage of being less expensive than the foreign varieties, and when once it has been used I do not think the purchaser will abandon it for any other. It can be obtained at the London Depôt, 12 Little Britain, or direct from Jersey.

### SUMMER SALES.

Messrs. Nicholson, of St. Paul's Churchyard, commence their summer sale on June 27th. Their catalogue shows some very pretty and inexpensive articles for ladies' wear, and they also supply nurses' uniforms.

The London Glove Company, 45 Cheapside and 82 New Bond Street, holds a sale commencing on June 29th.

### Appointments.

[No charge is made for announcements under this head, and we are always glad to receive, and publish, appointments. The information, to insure accuracy, should be sent from the nurses themselves, and we cannot undertake to correct official announcements which may happen to be inaccurate. It is essential that in all cases the school of training should be given.]

Cinder Hill Small-pox Hospital.—Mrs. Mary J. Golden has been appointed nurse matron. She was trained at the Belvidere Hospital, Glasgow, and at Hull, and has since been senior charge nurse at Knightswood Hospital, Partick, Glasgow, and superintendent nurse at Houghall Hospital, Durham.

Dumfries and Galloway Royal Infirmary.—Miss Helen Gregory Smith has been appointed lady superintendent. She was trained at the Western Infirmary, Glasgow, where she has since been ward sister, night matron, and assistant matron.

Islington Infirmary.—Miss Gertrude Norman, Miss Lena Driver, and Miss Francis Barns have been appointed staff nurses. They were all trained at Islington Infirmary.

Isolation Hospital, Heswell, Cheshire.—Miss Ethel Bentley has been appointed sister. She was trained at the Salford Union Infirmary, where she has since been sister.

Queen's Jubilee Hospital, London.—Miss Ida Mackintosh has been appointed matron. She was trained at the Sunderland Infirmary, and has since been district nurse in North London, matron of the Royal Victoria Nursing Home, and superintendent of the District Nurses, Ascot, and superintendent of the Elder Cottage Hospital and Nurses Training Home, Govan.

Seamen's Hospital Society's Branch Hospital, Royal Victoria and Albert Docks.—Miss Margaret I. Bedell has been appointed dispenser. She was trained at the Pharmaceutical Society's School, Bloomsbury Square, and has passed both the minor and major examinations of that society. She also holds the dispenser's qualification of the Apothecaries' Hall. She was formerly dispenser at the Victoria Eye and Ear Hospital, Herefordshire.

### TRAVEL NOTES AND QUERIES.

By our Travel Correspondent.

Helensburgh and Glasgow (Petso).—Helensburgh is immediately opposite to Greenock and sufficiently near to Glasgow for business men to make it their home. Frequent fast trains go between the two places. It is a good centre for excursions to the Clyde coast resorts and Loch Lomond is easily reached. There are plenty of hotels at Helensburgh, such as the "Queen's" and the "Imperial." It is about 20 miles from Glasgow. In Glasgow itself hotels are plentiful The Alexandra 10s. per day, Bridge Street Station Hotel 7s. 6d. per day, Buchanan Street Hotel 6s. 6d. per day, Cranstons Waverley (temperance) about the same, and many others. The Balmoral (temperance) is rather near to the University.

### To Nurses.

We invite contributions from any of our readers, and shall be glad to pay for "Notes on News from the Nursing World," or for articles describing nursing experiences at home or abroad dealing with any nursing question from an original point of view according to length. The minimum payment is 5s. Contributions on topical subjects are specially welcome. Notices of appointments, letters, entertainments, presentations, and deaths are not paid for, but we are always glad to receive them. All rejected manuscripts are returned in due course, and all payments for manuscripts used are made as early as possible after the beginning of each quarter.

### Echoes from the Outside World.

### Movements of Royalty.

On Saturday the King and Queen visited Wellington College, and Speech Day 1904 will always be remembered for the number of illustrious personages who assembled within the walls of the famous institution. One of the features of the day was the presentation of the Prince Consort history prizes by the Duke of Connaught; but the most inspiring presentation was, of course, that of the King's medal to J. R. Parsons. The winner, having kissed the King's hand kneeling, received the medal, and his Majesty addressed to him a few gracious words in an undertone, after which the King delivered a short speech without notes. Having expressed to Dr. Pollock, the master of the College, his very best thanks for the hearty welcome given him, his Majesty went on to express the hope that the boys at Wellington would always carry before them the memory of the great English commander who obtained a victory on June 18th, nearly ninety years ago. Subsequently, the King and Queen planted two trees at the end of the avenue, and the King inspected the guard of honour.

### The War between Russia and Japan.

There was a great battle in the vicinity of Wa-fang-tien last week, resulting in the complete defeat of the Russians. On Tuesday the fighting was mainly an artillery action, but on Wednesday the Japanese undertook an enveloping movement near Telissu, and after severe fighting routed their opponents, who hastily retreated northward. The Japanese commander reports the capture of colours, 14 quick-firing guns, and about 300 prisoners. The Japanese losses are estimated at under 1,000. General Oku reports that 1,516 Russian corpses were buried by the Japanese up to Friday evening last near Telissa, and the total Russian losses in the battle are estimated at several thousands. The Russian commander admits that his troops were obliged to fall back in presence of the largely reinforced columns of the Japanese. The acting Minister of Marine in Russia has received a despatch from Admiral Alexeieff to the effect that the work of repairing the warships at Port Arthur has been brought to a most successful conclusion.

### Endowment of Scientific Research.

Mrs. Percy Sladen, of Northbrook Park, Devonshire, has generously undertaken to devote the sum of £20,000 to the promotion of scientific research, particularly in the subjects in which her late husband, Mr. Walter Percy Sladen, sometime secretary and vice-president of the Linnean Society, was chiefly interested. She proposes to assign this sum, under the name of the Percy Sladen Memorial Fund, to certain trustees, in the first place of her own appointment, who are directed to employ the income arising therefrom, in their uncontrolled discretion, to any research or investigation in natural science, and more especially in the sciences of zoology, geology, and anthropology. They are also empowered, if they choose, to accumulate the income for the purpose of fitting out, or assisting to fit out, any expedition desired to further such research.

### Murder of the Governor-General of Finland.

General Bobrikoff, Governor-General of Finland, was entering the Senate at Helsingfors on Thursday, last week, when he was fired at with a pistol by the son of Senator Schaumann. Two shots were fired, and both took effect. One inflicted a serious wound in the stomach, while the other caused a slight injury in neck. The General received every attention, and an operation was successfully carried out, but he died on Friday. Nicolai Ivanovich Bobrikoff, who was regarded as one of the ablest men in modern Russia, had held positions of the Chief of the Staff of the Imperial Guard and of the St. Petersburg military district. He was aide-de-camp to the Czar and a member of the Military Advisory Board. His assassin, Eugene Schaumann, who committed suicide directly after he had perpetrated his crime, was an official of the Finland Education Department and Secretary of the Finnish Hunting Association. He had practised revolver shooting for several years. Recently, he had been suffering from melancholia. Mme. Bobrikoff has received telegrams of sympathy from the Czar, the Czarita, and the Metropolitan of St. Petersburg. Count Alexis Pavloveff Ignatieff, formerly Governor-General of the Province of Kieff, has been appointed Governor-General of Finland.

### Burning of an American Excursion Steamer.

On Wednesday, last week, an appalling disaster occurred in New York Harbour. The pleasure steamer *General Slocum* was conveying nearly 2,000 Sunday-school excursionists along the East River when a fire suddenly broke out. The vessel, hemmed in by the rocks of Hell Gate—otherwise Hurl Gate—was unable to turn, and the captain had to drive her forward at full speed in order to beach her, but this proceeding caused the flames to reach aft. A fearful scene of panic ensued, resulting in a terrible number of deaths by burning, crushing, or drowning. About 1,000 lives were lost, although, owing to the bravery of a few, many were saved who would otherwise have perished. One big stalwart man—most of the passengers were women and children—stood on the starboard paddle-wheel box of the burning boat and threw, one after another, 28 children into a net held by strong hands on one of the small boats. After throwing the last he was seen to stagger and fall backwards into the flaming pile. One boy of 14 was drowned after swimming ashore with four children. Another, a bricklayer, went out from the bank five times, rescuing a woman or child each time. The crew of the steamer got out the fire hose, but it was rotten; the lifeboats were fastened down by wire and could not be moved, and the life-belts sank like stones, dragging the wearers down with them. They were found to be largely composed of sodden cork and glue.

### New Play by Max Pemberton.

At Wyndham's Theatre a new play by Mr. Max Pemberton—his maiden effort as a dramatist—has been successfully produced. "The Finishing School" is a charming little love story, diversified with pleasant humour. The heroine runs away to Gretna Green, is caught by her guardian, Sir John Vane, and sent to a finishing school for a year. Here she hears that her lover's regiment is ordered to the war, and she breaks out of school to bid him good-bye, donning a boy's costume for the purpose. Her many adventures have a happy end, and the part is charmingly played by Miss Annie Hughes, Mr. Ben Webster, Mr. J. H. Barnes, and Mr. Frank Cooper also rendering good service.

### Women Writers at Dinner.

On Tuesday evening Miss Beatrice Harraden presided at the annual dinner of the Women Writers, and about 200 ladies were present. Miss Harraden proposed the health of their Majesties, and other speeches were delivered by Mrs. Sydney Webb, who explained "How the novelist prompted the economist," and by Mrs. de la Pasteur, who, recalling the fact that Jane Austen threw a handkerchief, when visitors called, over the manuscript on which she was engaged, said that the days were past when women writers need blush for their calling.

### Notes and Queries.

**FOR REGULATIONS SEE PAGE 143.**

*Midwifery.*

(97) 1. Can you tell me of any lying-in hospital where I could train in midwifery and study for the L.O.S. in return for my services? I have had two years' experience in general and three years' in private and district work, but I cannot afford to pay the whole fee. 2. Can you give me the address of Queen Victoria's Jubilee Institute for Nurses?—*I. R.*

1. You might write to the matrons of the Glasgow Maternity Hospital, 37 North Portland Street, Glasgow, or the Dundee Royal Infirmary, and ask if they can quote special terms to trained nurses as do the London Hospitals. But it is a long way for you to come from Northumberland. 2. Apply to the General Superintendent, Queen Victoria's Jubilee Institute for Nurses, 120 Victoria Street, S.W.

I am desirous of training for midwifery but wish to study beforehand. Could you kindly tell me what books would be best for me to get?—*R. B.* (Fulham).

If you write to the Secretary, the Association for Promoting the Training and Supply of Midwives, 12 Buckingham Street, Strand, W.C., you will receive the best advice.

Is there any institute at Portsmouth where pupils are taken for midwifery or monthly nursing?—*Hope.*

See reply to *R. B.*

*Maternity Fee.*

(98) I was engaged as maternity nurse for a certain date, and in consequence had to refuse other cases at a higher fee. My patient required me a month later, and I could not attend. After some correspondence, in which I told the husband that it was usual to pay half fees in such cases, he wrote to me that he was not liable and could not afford to pay. I know that he is in a fair position, and I have lost about £9 9s. Would you kindly tell me how I could recover my fee?—*A Constant Reader.*

Consult a solicitor. It would be a great advantage to you in such matters to belong to the Midwives' Institute, 12 Buckingham Street, Strand, W.C.

*Treatment.*

(99) Will you kindly tell me where a lady with limited means could receive electricity and massage for paralysis, either in London or Manchester?—*Nurse.*

The Hospital for Epilepsy and Paralysis, 4 Maida Vale, N.W., receives a few private patients.

Will you kindly tell me where a patient could receive the Weir-Mitchell treatment for as small a sum as a guinea a week?—*Hertford.*

You had better advertise. There is no public institution for the treatment.

*Hospital Training.*

(100) Will you kindly tell me if there are any general hospitals in London where probationers are trained for nursing?—*F. P.*

Yes, many. See "The Nursing Profession: How and Where to Train" for full list.

Are there any children's hospitals where probationers of 20 are received?—*F. P.*

Yes, a few.

Will you kindly tell me the earliest age at which a girl can begin to train as a hospital nurse? Is there a convalescent home where in return for services a girl could be gaining experience until it is time to begin training?—*E. M. G.*

Most hospitals prefer their probationers to be 23. A few hospitals receive them at 20, and one or two at 19. You might hear through advertisement of a small hospital or home where the girl could be employed as you suggest, but most matrons prefer probationers without previous training.

**Handbooks for Nurses.**

"Nursing Profession: How and Where to Train." 2s. net.; 2s. 4d. post free.

"Nurses' Dictionary of Medical Terms and Nursing Treatment. By Honnor Morten. 2s. post free.

"A Handbook for Nurses." By J. K. Watson, M.D. 5s. net; 5s. 4d. post free.

"Practical Guide to Surgical Bandaging and Dressings." By Wm. Johnson Smith, F.R.C.S. 2s. post free.

"The Examination of Urine." By J. K. Watson, M.D. 1s. net; 1s. 1d. post free.

"Case-book for Private Nursing." 3d. net; 4½d. post free per copy; or per doz. 3s., post free.

The above works are obtainable of all booksellers, or direct from The Scientific Press, Limited, 28 & 29 Southampton Street, Strand, London, W.C.

### For Reading to the Sick.

ACT, AND TRUST.

Sometimes trustful, often fearful,
In this world of shifting wrong;
Sometimes joyful, often tearful,
Still be this our rallying song—
Aye, in sadness
And in gladness,
Nobly act, for God is strong.

When oppressed by deep soul-sorrow,
Life beneath the darkest skies
Seems so drear that no to-morrow
Holds a threat of worse surprise—
In such sadness
As in gladness
Nobly act, for God is wise.

*Mackenzie Bell.*

We receive everything, both life and happiness; but the manner in which we receive, this is what is still ours. Let us humbly accept from God even our own nature, and treat it charitably, firmly, and intelligently. Let us accept ourselves in spite of the evil and disease.

Watch, disciple of life, watch and labour towards the development of the angel within thee.

Human life is but the preparation and means of approach to the spiritual life. The divine life is a series of successive deaths, in which the mind throws off its imperfections and its symbols, and yields to the growing attraction of the ineffable centre of gravitation, the Sun of intelligence and love.

To become divine is the aim of life; then only can truth be said to be ours beyond the possibility of loss, because it is no longer outside us, not even in us, but we are it, and it is we: we ourselves are a truth, a will, a work of God. Liberty has become nature; the creature is one with its creator—one through love.

The eternal life is eternally re-won. The eternal life is not the future life: it is life in harmony with the true order of things—life in God. We must learn to look upon time as a movement of eternity, as an undulation in the ocean of being. To live so as to keep this consciousness of ours in perpetual relation with the eternal is to be wise to live, so as to personify and embody the eternal, is to be religious. Spirit is Lord of matter, the world belongs to God. "Be of good cheer," saith a heavenly voice, "I have overcome the world."—*Amiel.*

What does it matter though the life below
For mortals prove unequal on the whole?
Flying and fading, wheresoe'er you go,
Have you not still above it all—your soul?
Your soul, which now reposes pure and clear,
Soon by its heavenly nature shall arise;
And bear you far beyond all sorrows here
And farther still beyond all murmuring sighs.

*J. S. B Monsell.*

